# The VACS Index Accurately Predicts Mortality and Treatment Response among Multi-Drug Resistant HIV Infected Patients Participating in the Options in Management with Antiretrovirals (OPTIMA) Study

**DOI:** 10.1371/journal.pone.0092606

**Published:** 2014-03-25

**Authors:** Sheldon T. Brown, Janet P. Tate, Tassos C. Kyriakides, Katherine A. Kirkwood, Mark Holodniy, Joseph L. Goulet, Brian J. Angus, D. William Cameron, Amy C. Justice, for the OPTIMA Team

**Affiliations:** 1 James J. Peters VA Medical Center, Bronx, New York, United States of America; 2 Department of Medicine, Icahn School of Medicine at Mount Sinai, New York, New York, United States of America; 3 Section of General Internal Medicine, VA Connecticut Healthcare System, West Haven, Connecticut, United States of America; 4 Yale University Schools of Medicine and Public Health, New Haven, Connecticut, United States of America; 5 VA Cooperative Studies Program Coordinating Center, VA Connecticut Healthcare System, West Haven, Connecticut, United States of America; 6 VA Palo Alto Healthcare System, Palo Alto, California, United States of America; 7 Department of Medicine, Stanford University School of Medicine, Palo Alto, California, United States of America; 8 Nuffield Department of Medicine, Oxford University, Oxford, United Kingdom; 9 University of Ottawa at the Ottawa Hospital Research Institute, Ottawa, Ontario, Canada; Institut national de la santé et de la recherche médicale (INSERM), France

## Abstract

**Objectives:**

The VACS Index is highly predictive of all-cause mortality among HIV infected individuals within the first few years of combination antiretroviral therapy (cART). However, its accuracy among highly treatment experienced individuals and its responsiveness to treatment interventions have yet to be evaluated. We compared the accuracy and responsiveness of the VACS Index with a Restricted Index of age and traditional HIV biomarkers among patients enrolled in the OPTIMA study.

**Methods:**

Using data from 324/339 (96%) patients in OPTIMA, we evaluated associations between indices and mortality using Kaplan-Meier estimates, proportional hazards models, Harrel’s C-statistic and net reclassification improvement (NRI). We also determined the association between study interventions and risk scores over time, and change in score and mortality.

**Results:**

Both the Restricted Index (c = 0.70) and VACS Index (c = 0.74) predicted mortality from baseline, but discrimination was improved with the VACS Index (NRI = 23%). Change in score from baseline to 48 weeks was more strongly associated with survival for the VACS Index than the Restricted Index with respective hazard ratios of 0.26 (95% CI 0.14–0.49) and 0.39(95% CI 0.22–0.70) among the 25% most improved scores, and 2.08 (95% CI 1.27–3.38) and 1.51 (95%CI 0.90–2.53) for the 25% least improved scores.

**Conclusions:**

The VACS Index predicts all-cause mortality more accurately among multi-drug resistant, treatment experienced individuals and is more responsive to changes in risk associated with treatment intervention than an index restricted to age and HIV biomarkers. The VACS Index holds promise as an intermediate outcome for intervention research.

## Introduction

In the past, CD4 count and HIV-1 RNA were successfully used as surrogate markers in HIV intervention research. They are strongly associated with the central pathophysiology of HIV infection and, among those not exposed to combination antiretroviral therapy (cART), were highly predictive of AIDS-defining events and death. They continue to be useful for evaluating new antiretrovirals because they are directly linked to suppression of HIV replication and can be monitored as continuous variables indicating response to treatment [1], [2]. However, in the modern ART era these markers alone inadequately respond to the range of illness that most commonly affects HIV infected patients.

CD4 count and HIV-1 RNA do not provide a comprehensive assessment of disease burden. This issue was nicely illustrated by the SMART and ESPRIT studies in which substantially more non-AIDS events than AIDS events were observed; most non-AIDS events were not correlated with CD4 and HIV-1 RNA levels [3]–[5]. More recent meta-analyses of randomized clinical trials show that prediction of AIDS events and death by CD4 and HIV-1 RNA is unreliable for people on cART and unsuitable for comparing treatment regimens for long-term clinical efficacy [6]. Further, recent cohort analyses show poor correlation between non-AIDS clinical outcomes and traditional biomarkers, further limiting their usefulness when evaluating management strategies concerned with long term clinical outcomes [7].

The Veterans Aging Cohort Risk Index (VACS Index) offers an alternative approach combining commonly collected HIV and “non-HIV” clinical biomarkers into a cumulative index weighted according to the risk of all-cause mortality. The VACS Index was developed in HIV infected US Veterans and validated in several European and North American cohorts [8]–[10]. It predicts both HIV and non-HIV related mortality including cardiovascular mortality [11] and it incorporates age, eight routine clinical laboratories, namely: CD4 cell count, HIV RNA, hemoglobin, aspartate aminotransferase (AST), alanine aminotransferase (ALT), platelet count, creatinine, and hepatitis C serologic status. The VACS Index more accurately discriminates mortality risk than a Restricted Index including only age, CD4 count and HIV-1 RNA. The VACS Index has yet to be evaluated in the context of a randomized trial or among patients with very advanced HIV disease. Most importantly, the responsiveness of the VACS Index to treatment intervention has yet to be evaluated and this is essential if the index is to be used to monitor treatment response or as an intermediate outcome in clinical research.

Options In Management with Antiretrovirals (OPTIMA) was a randomized trial of alternative treatment strategies for patients with advanced multi-drug resistant AIDS [12]. The advanced stage of HIV infection and extensive treatment experience distinguish the OPTIMA cohort from populations used for development and validation of the VACS Index, which evaluated subjects newly initiating cART [8], [10], [13]. Using data collected prospectively during the study, we evaluated the predictive accuracy of the VACS Index for all-cause mortality in OPTIMA and compared its performance with an index restricted to age and conventional HIV biomarkers [6], [14], [15]. We also compared on-study responsiveness to changes in risk associated with treatment interventions.

## Methods

### OPTIMA

The design and major outcomes for the OPTIMA study are reported in detail elsewhere [12], [16]. Briefly, OPTIMA was a multi-national collaboration (US Department of Veterans Affairs, Canadian Institutes for Health Research, UK Medical Research Council) conducted from 2001–2007. Of 368 enrolled patients with advanced multi-drug resistant HIV infection, 59% had past or present AIDS, median CD4 count was 110 cells/mm^3^, mean log_10_ HIV RNA was 4.74 copies/ml and mean number of potentially active antiretroviral drugs (ARV) by phenotypic susceptibility score = 1.5. A total of 165 (44.8%) of 368 subjects experienced a primary outcome. This included 67 deaths without a preceding AIDS defining even (ADE), and 98 ADE of whom 61 died subsequently. Of the 165 primary outcomes, the most common were death (40.9%), esophageal candidiasis (18.3%), *Pneumocystis jirovecii* pneumonia (PCP) (8.5%), cytomegalovirus disease (4.8%), HIV wasting syndrome (4.3%), *Mycobacterium avium* complex infection (3.7%), Kaposi’s sarcoma (2.4%), and cryptococcosis (2.4%). Fifty percent of patients developed 481 non-HIV-related serious adverse events (SAE), the major secondary clinical outcome. There was no significant difference in number or time to first non-HIV related SAE between Standard and Intensive ARV retreatment (log-rank p = 0.92), between Interruption and Continuation (p = 0.68) or across the four treatment approaches (p = 0.55). Vital status in OPTIMA was known for 99.7% of enrolled patients at the end of study. The endpoint review committee adjudicated 52% of deaths to be HIV-related, 2% due to ART medication, 15% unrelated to HIV or ART, and 31% as unattributable.

The 339 subjects contributing to the present analysis comprised the subset of OPTIMA who were randomized into the full 2×2 factorial design, either to an optimized standard ARV regimen (≤4 drugs, Standard) or treatment intensification (≥5 drugs, Intensification) with or without an initial 3 month treatment interruption (Interruption vs. Continuation).

### VACS Index

Prognostic factors in the VACS index include age, CD4 count, HIV-1 RNA, hemoglobin, AST, ALT, platelets, creatinine and HCV status. Composite markers of liver and renal injury (FIB-4 and estimated glomerular filtration rate (eGFR)) are computed. FIB-4, composed of AST, ALT, platelets, and age, is a validated indicator of liver fibrosis [17]. The eGFR, based on the Modification of Diet in Renal Disease (MDRD) equation, is a validated indicator of impaired renal function [18]. Data required for calculation of the VACS Index for subjects enrolled in OPTIMA were collected as part of routine protocol procedures at baseline and all subsequent study visits. When only one transaminase test was reported (per protocol) the other was estimated based on regression equations developed in the study sample. AST was estimated with ALT, age and HCV status. ALT was estimated with AST, age and hemoglobin. Components of the VACS Index and Restricted Index were categorized and assigned point values via a previously established system and summed to calculate a score (Table 1) [9]. Scores are typically between 0 and 100, with a higher score indicating worse prognosis.

**Table 1:** Point Values and Hazard Ratios for Death for Index Components.

	Points		Hazard Ratios for Death	
	Restricted Index	VACS Index	Restricted Index	VACS Index
**Age (years)**				
<50	0	0		
50 to 64	23	12	2.163	1.617
>65	44	27	4.315	2.976
**CD4 (cells/mm^3^)**				
>500	0	0		
350 to 499	10	6	1.47	1.375
200 to 349	10	6	1.333	1.176
100 to 199	19	10	1.869	1.508
50 to 99	40	28	3.837	3.036
<50	46	29	4.593	3.235
**HIV-1 RNA (copies/ml)**				
<500	0	0		
500 to 1×105	11	7	1.451	1.323
>1×105	25	14	2.308	1.78
**Hemoglobin (g/dL )**				
>14		0		
12 to 13.9		10		1.527
10 to 11.9		22		2.425
<10		38		4.665
**FIB-4**				
<1.45		0		
1.45 to 3.25		6		1.29
>3.25		25		2.715
**eGFR mL/min**				
>60		0		
45 to 59.9		6		1.274
30 to 44.9		8		1.393
<30		26		2.82
**Hepatitis C Infection**		5		1.216
**Theoretical maximum**	115	164		

FIB4: (years of age×AST)/(platelets in 10^9^/L×square root of ALT).

ALT: alanine transaminase.

AST: aspartate transaminase.

eGFR: 186.3×(serum creatinine^−1.154^)×(age^−0.203^)×(0.742 for women×(1.21 if black).

### Statistical Analyses

We first evaluated mortality from randomization (baseline). Observation time for survival analysis began at randomization and continued until death or end of study follow-up (December 31, 2012). We compared the association between risk score and mortality for the Restricted Index and VACS Index first using Kaplan-Meier (KM) plots stratified by quartile of baseline score rounded to the nearest five points. We then limited follow-up to 216 weeks to be consistent with the OPTIMA trial results and to approximate the period validated for the VACS Index. To translate scores to predicted mortality we fit a parametric (Gamma) regression model for all-cause mortality using risk index score as the only predictor. Mortality predictions at 216 weeks were compared graphically with observed mortality from KM estimates. For each five-point interval of score (collapsed if necessary to maintain at least 5 deaths and 10 survivors in each interval), a Kaplan-Meier mortality estimate and 95% confidence interval were calculated. Next we quantified the association between score and mortality using Cox models and calculated Harrell’s C statistic. Because C statistics are somewhat insensitive, we also calculated net reclassification improvement (NRI) to determine whether the VACS Index provided better discrimination than the Restricted Index [8], [9], [19], [20]. Groups of predicted risk made with approximately equal numbers of death using the Restricted Index were compared to the same cut-points using the VACS Index.

We also considered response to treatment between baseline and 48 weeks. To evaluate response to treatment interruption, we plotted mean index scores at baseline, 6, 12, 24 and 48 weeks. Scores for patients who died or were lost to follow-up were recorded as last value carried forward. To evaluate the association between change in risk score and subsequent mortality, we limited analysis to those alive at 48 weeks and used the difference in score between 48 weeks and baseline in a Cox model adjusted for baseline VACS Index score. We compared risk of death for those with the best 25% and worst 25% of risk score change to those in the central 50% of score change, for both the Restricted Index and the VACS Index. The analysis of risk score was restricted to 48 weeks for two reasons. First, the anticipated effect of treatment interventions on index scores due to the OPTIMA study interventions, whether due to treatment effect, toxicity, or clinical change in co-morbid conditions, was likely to be seen during the first one to two years following randomization. Subsequent changes in index scores are unlikely attributable to the study intervention. Second, one aim of the analysis was to evaluate the data as if either index was used as a more efficient surrogate for subsequent mortality. A 48 week trial of a treatment intervention is a practical interval over which to evaluate treatment outcomes. We compared baseline and 48 week risk index scores by intervention arm using linear regression with indicator variables for interruption and intensification.

We used SAS™ version 9.2 for all analyses, except calculation of Harrell’s c-statistic which used Stata™ version 11. In sensitivity analysis we excluded patients with estimated ALT and AST. Results were similar, so are not reported.

### Ethical Review

All patient data used in this analysis were collected as part of the OPTIMA protocol, which was reviewed and approved by independent ethics committees and institutional review boards and the trial was performed in accordance with the principles of Good Clinical practice and the Declaration of Helsinki. All patients gave written informed consent before any trial related procedure.

## Results

Of 339 patients evaluated, vital status at end of study was available for all but five, 308 had complete data for calculation of VACS Index at baseline and missing values were estimated for an additional 16 patients missing either baseline AST or ALT values. The remaining 15/339 patients without complete baseline VACS Index data were excluded.

Among 324 patients with known vital status and complete VACS Index included in subsequent analyses, mean baseline scores were 53.9 for the Restricted Index and 46.0 for the VACS Index (Table 2). Low CD4 counts and elevated HIV-1 RNA contributed less to overall score in the VACS Index than the Restricted Index.

**Table 2:** Mean Risk Index Scores at Randomization in 324 patients from OPTIMA.

	Patients per Category	Mean Score (SD)	
	Number (%)	Restricted Index	VACS Index
**Age (years)**		10.7 (12.9)	5.7 (7.1)
<50	184 (56.8)		
50 to 64	128 (39.5)		
≥65	12 (3.7)		
**CD4 (cells/mm^3^)**		27.2 (15.0)	16.8 (10.3)
≥500	2 (0.6 )		
200 to 499	83 (25.6)		
100 to 199	102 (31.5)		
50 to 99	44 (13.6)		
<50	93 (28.7)		
**HIV-1 RNA (copies/ml)**		16.0 (6.9)	9.5 (3.5)
<500	2 (0.6 )		
500 to 1×105	204 (63.0)		
≥1×105	118 (36.4)		
**Hemoglobin (g/dL)**			7.3 (8.6)
≥14	152 (41.7)		
12 to 13.9	130 (40.1)		
10 to 11.9	34 (10.5)		
<10	8 (15.7)		
**FIB-4**			6.5 (8.5)
<1.45	135 (41.7)		
1.45 to 3.25	138 (42.6)		
>3.25	51 (15.7)		
**eGFR mL/min**			0.7 (3.5)
≥60	305 (94.1)		
45 to 59.9	3 (0.9)		
30 to 44.9	11 (3.4)		
<30	5 (1.5)		
**Hepatitis C Infection**			1.2 (2.1)
Present	76 (23.5)		
Absent	148 (76.5)		
**Total Score**		53.9 (20.5)	42.0 (20.3)
Min-Max Range		21–115	13–110

FIB4: (years of age×AST)/(platelets in 10^9^/L×square root of ALT).

ALT: alanine transaminase.

AST: aspartate transaminase.

eGFR: 186.3×(serum creatinine^−1.154^)×(age^−0.203^)×(0.742 for women×(1.21 if black).

The median age at study enrollment was 48.6 years with a median follow-up on study of 3.9 years (IQR 2.5 years). Outcomes for the analyzed cohort were similar to those reported for the complete OPTIMA study [12]. No significant differences were found between interventions in the composite primary endpoint of time to all-cause mortality or new AIDS-defining event (ADE, 46% of subjects, p = 0.27), death (36%, p = 0.61), or ADE (27%, p = 0.25).

### Mortality from Baseline

**Figure 1:**
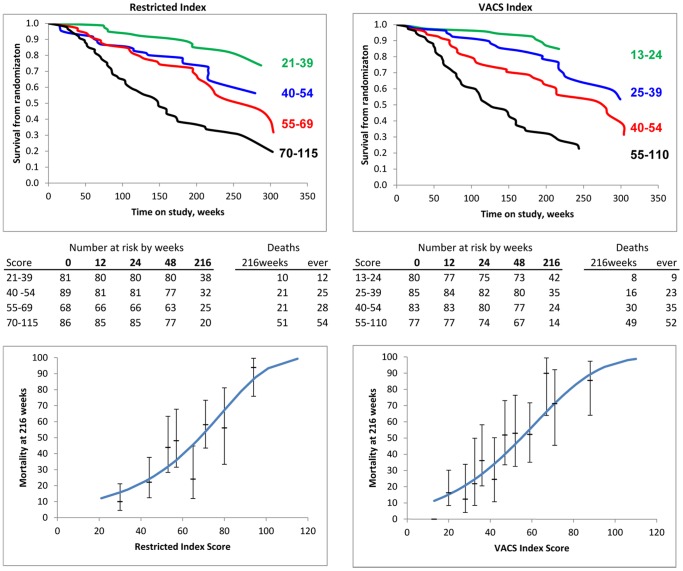
Mortality from Randomization Date by Risk Score. Left: Restricted Index; Right: VACS Index. Upper panels: Kaplan-Meier plots by quartile of score. Plot ends at last death. Lower panels: Mortality at 216 weeks vs. score, Lines: Predicted mortality, Points (95% Confidence Intervals) from Kaplan-Meier estimates using five-point intervals of score (collapsed if necessary to maintain at least 5 deaths and 10 survivors in each interval).

**Figure 2:**
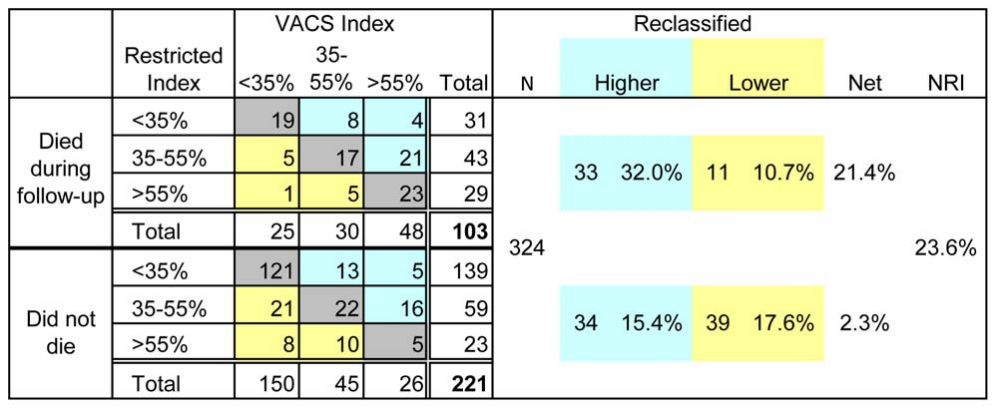
Net Reclassification Improvement (NRI) for Mortality at 216 weeks VACS Index versus Restricted Index. Groups of predicted risk were made with approximately equal numbers of death using Restricted Index compared to same cut-points using VACS Index. NRI is positive when more deaths have higher predicted risk and more survivors have lower predicted risk. A net 15.5% of those who died were reclassified to a higher risk using the VACS Index and 7.7% of those who lived were reclassified to a lower risk using the VACS Index.

There were 119 (36%) deaths in the analyzed cohort with 107 (33%) deaths at 216 weeks from baseline, the period included for survival analysis. The VACS Index had better prognostic accuracy than the Restricted Index. Although survival varied by baseline score for both indices (Figure 1, top) there was greater separation by quartile of risk score with the VACS Index. Predicted mortality was also better aligned with KM estimates using the VACS Index than the Restricted Index as shown by plots of mortality at 216 weeks versus score (Figure 1, bottom). With both indices, a five point increment of score was associated with 20% increased risk of death [VACS Index: HR 1.23 (95%CI 1.18, 1.29), Restricted Index: HR 1.22 (95% CI 1.16, 1.29). However, discrimination was greater with the VACS Index [c = 0.74 (95% CI 0.70, 0.79)] than the Restricted Index [c = 0.70 (95% CO 0.65, 0.75] p = 0.09. Compared with the Restricted Index, the additional variables included in the VACS Index resulted in a net reclassification improvement of 23% (Figure 2).

### Responsiveness

**Figure 3:**
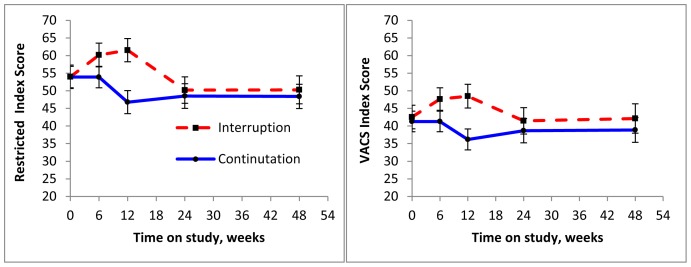
Risk Score by Time on Study (Weeks since Randomization) by Treatment Arm.

In those with treatment interruption, scores increased from baseline to 6 and 12 weeks (Figure 3). At 12 weeks there were significant differences between the continuation and interruption arms (with or without adjustment for baseline score): 14.7 points for the Restricted Index and 12.3 points for the VACS Index (p<0.001 for both). By week 24 there was no longer a significant difference by treatment arm.

Among those who had 12 weeks of treatment interruption similar proportions experienced changes in hemoglobin and FIB4 as with viral load but the direction of change differed. Specifically, 33% changed categories of VL with 26% moving to a higher level and 6% to a lower level. In contrast, equal proportions saw higher and lower hemoglobin levels (17% each, for an overall change of 34%) and 19% experienced higher (10% lower) FIB 4 levels. Once cART was re-initiated (12 weeks to 48 weeks) 46% achieved lower viral load levels (only 6% saw higher levels), whereas 19% developed worse hemoglobin levels (12% improved) and 15% developed worse FIB 4 scores (12% improved). Changes for eGFR were more modest for both treatment intervals with only 2% changing levels in the 12 weeks of treatment interruption and 6% (4% getter worse) changing after treatment was re-initiated.

For the 297/324 (92%) patients alive at 48 weeks, change in score between baseline and 48 weeks was associated with subsequent mortality (Table 3). Compared to those in the middle 50%, those with the best 25% and worst 25% change had significantly different risk of death. The association was a third stronger with the VACS Index [HR = 0.26 (95% CI 0.14, 0.49) for best response, HR = 2.08 (95% CI 1.27, 3.38) for worst response)] than with the Restricted Index [HR = 0.39 (0.22, 0.70) and 1.51 (0.90, 2.53)].

**Table 3:** Association Between Subsequent Mortality and Change in Score from Baseline (Randomization) to 48 Weeks, Adjusted for Baseline VACS Index Score.

		At Risk	Died	% Died	HR	LCL	UCL	p
Restricted Index Change								
	Baseline VACS Index (per 5 points)				1.30	1.23	1.37	<.0001
Best 25%	<−11	73	16	22%	0.39	0.22	0.70	0.001
Central 50% (Reference)	−11 to 0	164	49	30%	1.00			
Worst 25%	>0	60	21	35%	1.51	0.90	2.53	0.12
VACS Index Change								
	Baseline VACS Index (per 5 points)				1.33	1.26	1.40	<0.0001
Best 25%	<−11	74	13	18%	0.26	0.14	0.40	<0.0001
Central 50% (Reference)	−11 to 9	156	46	29%	1.00			
Worst 25%	>9	67	27	40%	2.08	1.27	3.38	0.003

Change in index score from baseline was assessed for 297/324324 patients surviving to 48 weeks. Risk of subsequent death was assessed by quartile with the central 50% serving as the reference group.

## Discussion

The VACS Index accurately predicted observed mortality rates in a randomized intervention trial showing better discrimination, calibration, and responsiveness than an index restricted to age and HIV biomarkers. Of note, the VACS Index was developed and validated among those within their first 5 years of ART exposure. In contrast, OPTIMA subjects were highly treatment experienced, yet the VACS Index displayed similar performance characteristics as was seen in other cohorts. The VACS Index shows promise as a candidate intermediate outcome for clinical research.

The superior mortality prediction using the VACS Index is significant and consistent with the observation that much of the expected high mortality and SAE rate in OPTIMA was not attributable to ADEs, nor were they adjudicated as HIV-related [10]. The design and development of OPTIMA anticipated that AIDS complications would dominate important clinical outcomes in a highly vulnerable population with limited treatment options. However, only 51% of deaths were attributed to AIDS or HIV associated events and there were 466 non-HIV related SAEs in 176 patients compared with 99 ADEs in 60 patients. Clinical events contributing to a substantial burden of patient morbidity were therefore not captured in the primary outcome measure selected for OPTIMA even though they undoubtedly contributed to overall mortality and may be associated with HIV disease progression. The improved prediction of mortality by the VACS Risk Index (over the Restricted Index) underscores its ability to utilize biomarkers embracing diverse, clinically relevant non-HIV conditions that are associated with SAEs and mortality while remaining responsive to the consequences of advanced HIV infection and ART toxicity.

If an intermediate outcome is to be useful in intervention research it must be responsive to changes in risk resulting from the intervention under study. As a randomized trial of HIV treatment, OPTIMA provides an excellent demonstration of the responsiveness of the VACS Index to clinical changes associated with treatment interventions. Both indices increased significantly after stopping cART reflecting expected increases in HIV RNA and decreases in CD4 count in the Interruption group. Differences in scores between Continuation and Interruption Groups were seen by the time of treatment resumption and these differences resolved by week 24 suggesting direct response to changing interventions. Thus, our finding that, compared with the Restricted Index, the VACS Index is responsive to changes after baseline and that changes in the VACS Index were more predictive of mortality after adjustment for baseline score suggests that the VACS Index also fulfills this requirement.

Specifically, in addition to the expected changes in CD4 count and HIV-1 RNA, hemoglobin, creatinine (part of eGFR), AST, ALT, and platelets (all three part of FIB-4) changed substantially during the intervention interval. While these improved overall, some individuals displayed incongruity between HIV and non-HIV biomarkers evidenced by the stronger association of change in VACS Index score with mortality. A significant advantage of the VACS Index is that it incorporates both likely benefits and harms of antiretroviral treatment by including both HIV biomarkers and markers of general organ system function. Of note, non-HIV organ system biomarkers were more discordant with HIV treatment than were HIV biomarkers suggesting that, for at least some patients, treatment toxicity may have muted the benefit of ART. The VACS Index therefore has the potential for reconciling, in a single outcome measure, the frequently opposing outcomes of therapeutic benefit versus adverse treatment effects.

It is also important to note that indices frequently fail to generalize to populations of differing severity of illness or at different points in their treatment regimen [21]. The original VACS Index cohort consisted of patients with moderately advanced HIV infection, median CD4 count = 281 cells/mm^3^, who were initiating treatment [13]. More than 50% of the population in subsequent multi-national cross-cohort evaluations of the Index had CD4≥350 cells/mm^3^ with more than 75% having HIV RNA ≤500 copies/ml. The contrasting OPTIMA cohort consisted of patients with clinically advanced AIDS, median CD4 count = 119 cells/mm^3^, highly treatment experienced, failing current treatment and having few effective anti-retroviral treatment options. Discrimination of mortality by the VACS Index (c-statistic = 0.74) was near that seen in the large validation cohorts (c-statistic = 0.78) and compares favorably with the prediction of cardiac events by the Framingham Risk Index [22]. Confirmation of predicted mortality by the VACS Index in OPTIMA validates and extends the generalizability of the VACS Index to an important, previously unexamined HIV infected population.

The VACS Index may help with practical issues in conducting intervention research. The VACS Index is composed of clinical biomarkers recommended as part of routine clinical management. The algorithm for calculating the score and translating it to a risk estimate is publically available (HTTP://VACS.MED.YALE.EDU) and the supporting evidence is readily available and updated (WWW.VACOHORT.ORG). The use of the VACS Index offers two additional advantages that could improve study efficiency and generalizability. First, study duration could be shorter as the need to follow for long term clinical events (mortality) is obviated. Second, the index could be used as a means of comparing the relative effectiveness of treatments to manage complex comorbidities shown to interact with HIV care such as diabetes [23], tighter control of lipids and other cardiac risk factors [24], reducing alcohol and illicit drug use [25].

There are potential limitations to this analysis. The VACS Index was developed from databases of the Veterans Health Administration and the majority of subjects in OPTIMA were veterans enrolled in care during the same interval. The cohorts in which the VACS Index was developed included patients recently initiating treatment so that there is unlikely to be overlap with the heavily treatment experienced veterans eligible for enrollment in OPTIMA. While our results demonstrate that the VACS Index performs well in a population distinct from the more general population in which it was developed, there may still be room for refinement of weighting of the VACS Index when considering specific populations. Adjustment of estimated weights to well defined subpopulations may be especially desirable if the Index is intended as a comparative outcome measure for treatment interventions. While the VACS Index was not conceived as an *a priori* measure in the design of OPTIMA, all components necessary for its calculation were collected as part of routine study procedures, thus reducing the risk of retrospective bias. It should be noted that premature assessment of the VACS Index responsiveness to interventions such as temporary treatment interruption may not be indicative of subsequent mortality. The design of future clinical trials using the VACS Index as a continuous variable will need to incorporate appropriate monitoring guidance to avoid such misinterpretations. Finally, treatment regimens employed during OPTIMA reflected the standard of care between 2001 and 2007, many of which are no longer employed, at least in resource rich environments. However, OPTIMA was a pragmatic trial of treatment strategies and the regimens used changed over its six year course as newer drugs became available and treatment preferences changed. ART preferences continue to evolve and there is increasing worldwide diversity in specific regimens employed. Since the VACS Index is a composite measure simultaneously embracing the benefits and toxicities of diverse treatments and co-morbidities, we have no reason to think that its responsiveness to treatment interventions is dependent upon the particular ART chosen. Consequently, we believe the insights gained from the analysis of OPTIMA remain relevant to contemporary treatment contexts.

Ultimately, determining whether clinical management is improved through the use of the VACS Index to help guide therapy will require a randomized trial. For now, the advantages of including the VACS Index as an intermediate outcome in clinical research appear compelling. This is especially true since these biomarkers are assayed in the course of routine clinical care and the means of calculating and interpreting the scores are publically available.
